# Plant Traits Guide Species Selection in Vegetation Restoration for Soil and Water Conservation

**DOI:** 10.3390/biology12040618

**Published:** 2023-04-19

**Authors:** Denggao Fu, Xiaoni Wu, Lianyu Hu, Xudong Ma, Chunjie Shen, Huaye Shang, Gongning Huang, Yongjian He, Changqun Duan

**Affiliations:** 1 Yunnan Key Laboratory for Plateau Mountain Ecology and Restoration of Degraded Environments, School of Ecology and Environmental Sciences, Yunnan University, Kunming 650091, China; ecodenggaofu@163.com (D.F.);; 2 Yunnan International Cooperative Center of Plateau Lake Ecological Restoration and Watershed Management, Kunming 650091, China; 3 School of Agronomy and Life Sciences, Kunming University, Kunming 650214, China; wuxiaoxiaoni@163.com

**Keywords:** plant functional response type, plant functional effect type, functional traits, soil and water conservation, ecological restoration

## Abstract

**Simple Summary:**

It is an important objective of ecological restoration to select suitable plant species in order to construct plant communities and achieve certain soil and water conservation capacities. Using species functional traits to construct a response-and-effect framework is a promising method for determining how species in changing environments will influence ecosystem functions. Here, we investigated plant functional traits, soil properties, and ecohydrological functions for the most common vegetation restoration types in mid-Yunnan, China, and used seven plant functional traits to identify seven plant functional effect types in relation to soil and water conservation capacity, and two plant functional response types to soil physicochemical properties. The results indicated that specific leaf area was the key trait not only for functional effect types, but also for functional response types. In addition, eight overlapping species between plant functional response types and functional effect types were selected as the key restoration species. These results provide a methodological guide for species selection for recovery, as well as a species inventory for the restoration of degraded ecosystems in this region.

**Abstract:**

Great efforts have been made to improve the soil and water conservation capacity by restoring plant communities in different climatic and land-use types. However, how to select suitable species from local species pools that not only adapt to different site environments, but also achieve certain soil and water conservation capacities is a great challenge in vegetation restoration for practitioners and scientists. So far, little attention has been paid to plant functional response and effect traits related to environment resource and ecosystem functions. In this study, together with soil properties and ecohydrological functions, we measured the seven plant functional traits for the most common species in different restoration communities in a subtropical mountain ecosystem. Multivariate optimization analyses were performed to identify the functional effect types and functional response types based on specific plant traits. We found that the community-weighted means of traits differed significantly among the four community types, and the plant functional traits were strongly linked with soil physicochemical properties and ecohydrological functions. Based on three optimal effect traits (specific leaf area, leaf size, and specific root length) and two response traits (specific leaf area and leaf nitrogen concentration), seven functional effect types in relation to the soil and water conservation capacity (interception of canopy and stemflow, maximum water-holding capacity of litter, maximum water-holding capacity of soil, soil surface runoff, and soil erosion) and two plant functional response types to soil physicochemical properties were identified. The redundancy analysis showed that the sum of all canonical eigenvalues only accounted for 21.6% of the variation in functional response types, which suggests that community effects on soil and water conservation cannot explain the overall structure of community responses related to soil resources. The eight overlapping species between the plant functional response types and functional effect types were ultimately selected as the key species for vegetation restoration. Based on the above results, we offer an ecological basis for choosing the appropriate species based on functional traits, which may be very helpful for practitioners involved in ecological restoration and management.

## 1. Introduction

Soil and water conservation is one of the most important ecological functions and services. Because of their influence on soil erosion, ecohydrology, and water quality, changes in land use or management have imposed great pressure on watershed ecological restoration and management [1,2]. The most efficient method to improve soil and water conservation abilities is thought to be the effective restoration of soil and water conservation forests [3,4]. However, current restoration strategies are mainly focused on monospecific vegetation restoration, which leads to poor soil and water conservation efficiency [5,6]. Therefore, it is necessary to understand how to select specific multiple species to maximize the soil and water conservation capacity of restored vegetation. 

Trait-based approaches are increasingly used to explore the responses to environmental changes and ecological effects on ecosystem processes and functions [7,8]. Recent research has shown that ecohydrological processes and functions mainly depend on the functional traits of living plants [9,10,11,12]. Plant traits not only relate to plant growth and persistence (e.g., resource acquisition and use, recruitment, and dispersal), which are response traits, but also determine ecosystem processes and functions—the effect traits [7,8,13,14,15,16]. Based on the plants’ response and effect traits, plant species can be classified into different plant functional types (PFTs), namely, plant functional response types (PF_re_Ts) and plant functional effect types (PF_ef_Ts). This provides an effective way to select suitable species that can not only cope with specific environmental conditions, but also improve ecosystem functions.

The differentiation between PF_re_Ts and PF_ef_Ts has been suggested in the literature [17,18]. It is assumed that PF_re_T is a group of plants with similar responses to a given environmental factor and that PF_ef_T is a group of plants with a similar effect on certain ecosystem variables [8]. If PF_re_Ts and PF_ef_Ts are similar or closely correlated, the effects of environmental change on the community functional structure may potentially spread to the ecosystem functions. Conversely, if PF_re_Ts and PF_ef_Ts are distinct, communities may be able to withstand the effects of environmental changes while still maintaining ecosystem functions, despite changes in community functional structure. Lavorel and Garnier demonstrated that traits related to nutrient gradients and those affecting ecosystem functions overlapped highly [8], whereas Poorter and De Jong discovered that traits associated with response to fire and those affecting flammability had little overlap [19]. Therefore, the degree of overlap and correlation between PF_re_Ts and PF_ef_Ts appears to be useful for identifying key restoration species and exploring the relationships between the response of community to environmental disturbance and the effect on ecological functions [15]. However, there are little empirical data to support the presence of these relationships using plant functional traits.

The Central Yunnan Plateau is a crucial region for soil and water conservation in the Yangtze River Basin. Previous research has shown that plant attributes (e.g., height, growth type, and specific leaf area) are close to ecohydrological functions [9], which suggests that these attributes could be used as functional effect traits. In addition, many studies have found that some functional traits, e.g., specific leaf area, leaf nitrogen concentration, leaf dry matter content, and plant height, can be selected as functional response traits to soil resources under different soil and water loss conditions [7,8,10,11,15,16]. Here, we firstly investigated the distribution pattern of functional traits and the dynamics of soil resources and the soil and water conservation capacity. Then, an optimization algorithm was then performed to identify the PF_re_F to site resources and PF_ef_F in relation to the soil and water conservation capacity for the common species of the Central Yunnan Plateau. The main aims in this context are (i) to compare the differences in plant traits, soil properties, and ecohydrological functions across different vegetation restoration types; (ii) to examine the relationships between functional traits and environmental factors and ecohydrological functions; and (iii) to identify functional traits for choosing species of PF_re_Ts and PF_ef_Ts for revegetation of soil erosion area and control of water and soil loss.

## 2. Materials and Methods

### 2.1. Study Area

Field work was done in Mouding County (25°24′09′′ N; 101°28′18′′ E), which is about 200 km west of Kunming, Yunnan Province, China. The region has a warm temperate zone (Köppen–Geiger climate classification), with an average annual rainfall of 846 mm. The rainy season runs from May to October. The soil of the area is Cambisols (FAO/UNESCO classifications). A subtropical evergreen broad-leaved forest served as the area’s primary vegetation, but it has now been nearly entirely destroyed. Since the 1980s, some of the area has been replanted with fast-growing *Pinus yunnanensis* after deforestation. Other remnant coppices and pastures were abandoned and formed different secondary stands. This area currently includes secondary shrubland (SL), *Pinus yunnanensis* coniferous forest (CF), mixed needle–broad-leaved forest (MF), and natural secondary forest (NSF). These four main vegetation restoration types were selected to investigate the species traits, soil properties, and ecohydrological functions.

### 2.2. Investigation and Monitoring Procedures

Five non-contiguous duplicate plots were randomly assigned for each plant community type. All 20 plots had similar climatic conditions, soil types, and slope gradients. All of the woody species (height > 30 cm) were identified and counted. The species relative abundance was calculated based on the diameters at the breast height or basal area.

Seven representative plant functional traits were evaluated for the identification of the plant functional types, which are related to the plant structure, nutrients, and growth. The functional traits include the specific root length (SRL), leaf area (LA), leaf nitrogen concentration (LNC), leaf dry matter content (LDMC), specific leaf area (SLA), tree height (H), and plant growth type (GT). LNC, SLA, SRL, and LDMC were selected as the response traits relative to water and nutrients [20], and GT, H, LA, SLA, and SRL were used as the effect traits relative to water and soil conservation [9,10,11]. The traits of 27 common species were measured following standardized protocols [20]. Moreover, five random soil samples (between 0 and 30 cm depth) were collected in each plot. They were analyzed for the soil organic carbon (SOC), total nitrogen (TN), total phosphorus (TP), and pH. Three another undisturbed soil samples were also collected to measure the soil bulk density (BD) and soil water content (SWC) [21]. 

Three random plots from among the five plots were chosen to monitor the ecohydrological processes and functions. Two automatic rain gauges and two normal rain gauges were placed in an open area of the experimental station to monitor precipitation. Moreover, plastic collectors were put in place under the canopy of 10 sample trees to catch the throughfall. Because of the extremely low percentage of stemflow, we combined canopy interception and stemflow in this study and used the following formula: I_n_ = P − T_f_, where I_n_ is the sum of canopy interception (I_c_) and stemflow (S_f_), and P and T_f_ represent precipitation and throughfall, respectively. The difference between the mass of dry material and wet material was used to calculate the maximal water-retaining capacity of litter (MWClitter). The formula used to determine the soil maximum water-retaining capacity (MWC_soil_): MWC_soil_ = 10,000 P_t_ h, where P_t_ is total porosity of soil and h is soil depth. We installed an automatic flow gauge to measure the amount of soil surface runoff along the lower edge of each plot. After each runoff occurrence, 500 mL of soil surface runoff was sampled and the soil erosion was measured by drying three replicates of 50 mL runoff at 105 °C. To create a new average volume, the soil surface runoff and soil erosion data from the previous four years were averaged. 

### 2.3. Statistical Analysis 

As the plant community’s functional diversity index, the mean species trait values and the species relative abundance were used to compute the CWMs for each trait and plot as follows [22,23]: ${CWM}_{ip}=\sum_{i=1}^{S}a_{ip}\times t_{i}$, where *a_ip_* represents the relative abundance of species *i* in plot *p*, *t_i_* represents the mean trait value of species *i*. Soil physicochemical properties, CWMs, and water and soil conservation parameters under different vegetation types were compared using one-way analysis of variance (ANOVA) and the Fisher’s least significant difference (LSD) test. In addition, we analyzed the correlations between the plant traits and soil properties and water and soil conservation parameters using Spearman’s correlation. 

PFTs were defined using the optimization algorithm and were implemented with SYNCSA software [24]. The algorithm used polythetic cluster analysis to define the PFTs and then looked for the most pertinent features in a recursive process. The degree of convergence between community variation and environmental factors served as the foundation for the optimization procedure. We employed the matrix correlation ρ(D;Δ) to quantify this. D is a dissimilarity matrix of plots computed from matrix X of PFTs by plots. Δ is a dissimilarity matrix of the same plots based on soil environmental data. ρ is defined as Pearson’s product-moment correlation. Larger ρ values suggest that the PFTs are more likely to be “functional” for the environmental factors considered [25]. Matrix E (matrix environment) includes two matrices: the soil resource matrix and the soil and water conservation matrix. In addition, we used a redundancy analysis to examine the relationships between PF_ef_Ts and PF_re_Ts across all of the communities. The performances of PF_ef_Ts and PF_re_Ts in each quadrat were taken as explanatory variables and response variables, respectively. Moreover, the overlapping species between PF_ef_Ts and PF_re_Ts were identified as the key restoration species for the construction of soil and water conservation forests.

## 3. Results

### 3.1. Plant Traits, Soil Properties, and Ecohydrological Functions

Different functional traits showed trait-specific responses to the community restoration types. SL and CF had a higher CWM for SLA, LNC, and SRL. MF and NSF had a higher CWM for LDMC, LA, and H (Table 1). For the soil physicochemical properties, SWC and SOC were higher in NSF and MF than in SL and CF. The soil in NSF had significantly higher TN and TP than in the other plant communities (Table 1). Moreover, significantly higher interception of canopy and stemflow, and considerably lower soil erosion and soil surface runoff were found in NSF. However, the maximum water-holding capacity of soil displayed a non-significant difference among the four plant communities (Table 1).

Spearman’s correlation between the plant functional traits and soil basic properties and ecosystem function indices are shown in Table 2. The results display that the CWM of all traits except LNC was significantly linked to the soil properties (SWC, BD, and SOC) and I_n_. Variations in the CWMs for SLA and SRL were significantly associated with soil TP, soil surface runoff, and soil erosion. Moreover, there were close negative relationships between soil erosion and CWMs for LA and H (Table 2).

### 3.2. Plant Functional Response Types (PF_re_Ts) Related to Soil Resources

Based on the four selected traits and with a maximum congruence between species traits and soil variation (ρ = 0.18), a subgroup of two traits (SLA and LNC) defined two plant functional response types (PF_re_Ts) (Table 3). Species with a higher LNC and lower SLA were grouped into PF_re_Ts, such as *Lithocarpus polystachya*, *Eurya nitida*, *Osteomeles schwerinae*, and *Eucalyptus smithii*. Grouped in PF_re_T-2 were those species with a lower SLA and LNC. The most representative species were *Keteleeria evelyniana*, *Ternstroemia gymnanthera*, *Pyrus pashia*, and *Myrsine africana*. The presence of these two PF_re_Ts was 100%, but the average performance of PF_re_T-1 was two times greater than that of PF_re_T-2.

### 3.3. Plant Functional Effect Types (PF_ef_Ts) Related to Water and Soil Conservation

Seven plant functional effect types (PF_ef_Ts) defined by three optimal traits (SLA, LA, and SRL) were found with a large congruence ρ(D, Δ) of 0.40. Grouped in PF_ef_T-1 were those species with a higher SLA and SRL, despite the lowest LA. The most representative species was *Keteleeria evelyniana*. Grouped in PF_ef_T-2 were species with a lower SLA, SRL, and LA, such as *Cyclobalanopsis glaucoides* and *Pinus yunnanensis*. Plant type PF_ef_T-3 included those species with the lowest SLA and LS and the lowest SRL (e.g., *Pyrus pashia* and *Myrica nana*). PF_ef_T-4 was the species with the highest SRL and the lowest LS, similar to *Myrsine africana*. The first four PF_ef_Ts had higher presence values, and the average performance of PF_ef_T-2 (Avg. Perf. = 15.5) was the highest among the seven functional types (Table 4).

### 3.4. Evaluating the Relationships between PF_re_Ts and PF_ef_Ts

The relationship between the community composition given by PF_ef_Ts associated with ecological functions given by PF_re_Ts associated with nutrient loss was further evaluated by redundancy analysis (Table 5). The results showed that the sum of all canonical eigenvalues (PF_ef_Ts) only explained 21.6% of the variation in the response matrix (PF_re_Ts). Meanwhile, the values of all canonical eigenvalues were smaller than the first non-canonical eigenvalues (Table 5). These results indicate that community functional effects on soil and water conservation could not explain the overall changes in community response after soil nutrient loss.

### 3.5. Identification of Key Restoration Species

**Figure 1:**
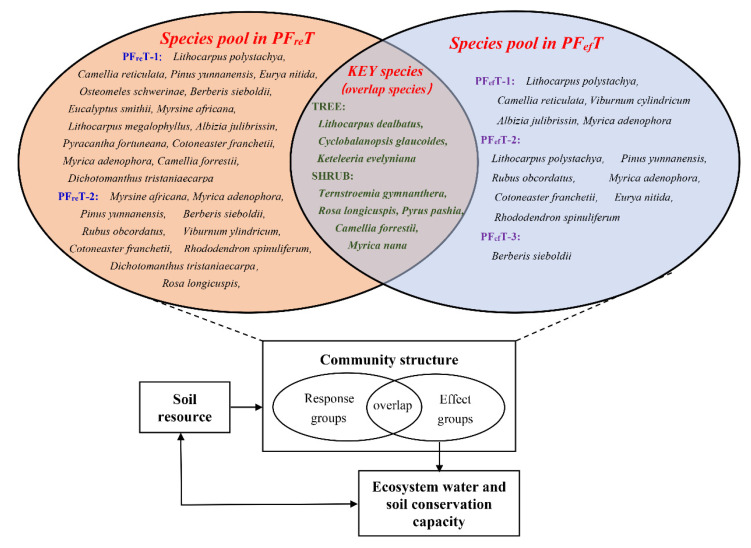
Species pools of PF_re_Ts and PF_ef_Ts and the identification of key restoration species using a trait-based response-and-effect framework.

On the basis of the identification of plant functional types, we found eight overlapping species, including three tree species (*Cyclobalanopsis glaucoides*, *Lithocarpus dealbatus*, and *Keteleeria evelyniana*) and five shrub species (*Camellia forrestii*, *Ternstroemia gymnanthera*, *Pyrus pashia*, *Myrsine nana*, and *Rosa longicuspis*) (Figure 1). These overlapping species could be used as the key species for vegetation restoration to adapt to the site soil conditions and maintain local water and soil conservation capacity.

## 4. Discussion

### 4.1. Selection of Plant Functional Traits

Our results have shown that SLA is the better trait for optimally defining PF_ef_Ts and PF_re_Ts. Functional traits optimally defining PF_ef_Ts were SLA, SRL, and LA. SLA and LA had significant effects on the water holding capacity of leaves and the rainfall interception capacity of forest canopies [9], and SRL improved ecohydrological functions by altering the maximum water-retaining capacity of soil [9,26]. In the identification of PF_re_Ts, SLA and LNC were the better traits for optimally defining PF_re_Ts. It has been confirmed that SLA and LNC are better response traits in many studies [15,19,27,28]. In addition, the maximum congruence ρ(D, Δ) of 0.18 was not high, and only two types were defined in the identification of PF_re_Ts, which suggests that representative data on LNC, SLA, SRL, and LDMC are not sufficient for the identification of PF_re_Ts and that more response traits should be selected for defining PF_re_Ts.

### 4.2. Identification of Plant Functional Effects and Response Types

Seven plant functional effect types (PF_ef_Ts) defined by three optimal traits (SLA, LA, and SRL) were found with a maximum congruence. The results showed that the first three PF_ef_Ts had lower leaf areas, which was not consistent with the fact that the larger the leaf area was, the higher the rainfall interception of the leaf and canopy and the lower the water and soil loss. These results may suggest that leaf area was not the main factor controlling soil and water loss. Canopy, litter layer, and root-bearing soil were found to have important roles in water and soil conservation. SLA was an effective indicator of rainfall interception by the canopy. At the same time, SLA was also an indicator of litter decomposition. Leaves with higher SLA always had faster decomposition rates, resulting in lower litter mass accumulation and soil and water conservation capacities. Species with higher SRL would be important for increasing the maximum water-retaining capacity of soil and reducing the soil surface runoff and soil erosion by root nets [10]. However, the reason for two PF_ef_Ts in the first three PF_ef_Ts with medium SLA and SRL may be explained by the patterns of trade-off between the growth rate and cumulative biomass because plants with a higher SLA and SRL always have a higher growth rate, but have lower cumulative biomass. Grouped in PF_ef_T-3 were the understory shrub species with the lowest SLA and lower SRL. Shrub species affected rainfall interception and soil erosion by the shrub canopy layer and surface root-bearing soil.

In the identification of plant PF_re_Ts, the two PF_re_Ts had lower SLAs, suggesting that the species with lower SLAs may be better in sites with poor nutrients due to severe soil and nutrient loss. The difference in LNC between the two PF_re_Ts showed that different strategies were adopted. PF_re_T-1, with a higher LNC, exhibited an “extravagant nutrient use strategy” in relatively rich soil nutrient conditions, while PF_re_T-2 had the opposite strategy (“conservative nutrient use strategy”), such as a lower LNC and high N resorption efficiency from senescing leaves [9,20].

### 4.3. Implications of Plant Functional Types for the Construction of Soil and Water Conservation Forests 

Achieving efficient soil and water conservation in the Central Yunnan Plateau is the key concern for forest managers and ecologists [4,29]. Maintaining the local species pool by active planting is frequently the first step in restoration practices. The stressful soil, water, and nutrient conditions in Central Yunnan, however, pose a serious threat to the effectiveness of community restoration [5]. Thus, soil surface runoff and soil erosion must be successfully reduced in the restored sites [30]. Our study proposes choosing mid-successional species for restoration efforts, particularly large shrubs and trees, to resolve these two seemingly incompatible properties. This method is derived from the trade-offs between plant responses to stressful environmental conditions and the plant effects on ecosystem functions [15,16,17]. In the early stages of succession, although fast-growing species with high SLA, LNC, and SRL but low LDMC could hasten nutrient turnover time through a high litter decomposition rate, the resulting reductions in accumulated standing biomass, litter biomass, and fine root biomass did not successfully control soil surface runoff and soil erosion. On the contrary, slow-growing species in later phases of succession could retain specific ecohydrological processes and functions through high LAI, litter biomass, and fine root biomass, but they were unable to properly adapt to challenging environmental conditions (e.g., higher light, lower soil water content, and poor soil nutrient). Therefore, we believe that it may be preferable to introduce mid-successional species for vegetation restoration in Central Yunnan rather than late- or early-successional species. Firstly, the utilization of mid-successional species, the majority of which are drought- and infertility-tolerant forms with regeneration niches typically associated with open environments [31], could help to boost restoration success. Secondly, they tend to have higher SRL and deeper roots at the seedling stage, which is crucial for the establishment of earlier erosion protection [32]. Finally, they naturally quicken succession toward a mature community by facilitating the recruitment of species that are vulnerable to drought under their canopies. 

We used the response-and-effect trait framework to assess the overlap between PF_re_Ts and PF_ef_Ts and to identify key species in the subtropic mountain ecosystem. However, the limitations of this method should be considered by forest managers and ecologists. For example, interspecific relationships among the selected species were not considered in this method, considering that interspecific competition may affect the community functional structure or ecosystem functions. Further research into traits and interspecific relationships and tradeoffs would improve the accuracy of this method. Nevertheless, this research suggests that a trait-based method may help guide forest restoration and management to maintain ecosystem services.

## 5. Conclusions

In the present study, we demonstrated that plant functional traits can be used to construct a response-and-effect framework for determining how species in changing environments will influence ecosystem functions. Our results showed that different functional traits displayed trait-specific responses to community restoration types. Analyses of the correlations and optimization algorithm suggested that SLA was the key response and effect trait, and it could not only effectively respond to soil resources, but it also strongly affected soil ecohydrological functions. Based on three optimal effect traits (SLA, LS, and SRL) and two response traits (SLA and LNC), seven functional effect types in relation to soil and water conservation capacity (interception of canopy and stemflow, maximum water-holding capacity of litter, maximum water-holding capacity of soil, soil surface runoff, and soil erosion) and two plant functional response types to soil physicochemical properties were identified. Moreover, eight overlapping species between plant functional response types and functional effect types were selected as the key restoration species. These results provide a methodological guide for species selection for recovery, as well as a species inventory for the restoration of degraded ecosystems in this region.

## Figures and Tables

**Table 1:** Plant traits, soil physicochemical properties, and water and soil conservation capacity in four land restoration types.

	Shrubland	Coniferous Forest	Mixed Forest	Natural Secondary Forest
SLA	13.25 ± 0.24 b	17.47 ± 0.58 a	11.32 ± 0.61 c	9.71 ± 0.06 d
LDMC	285.7 ± 3.7 c	322.4 ± 7.3 b	395.8 ± 13.6 a	406.8 ± 11.9 a
LNC	14.02 ± 0.18 a	8.90 ± 0.17 b	8.74 ± 0.14 b	9.22 ± 0.10 b
SRL	38.90 ± 0.69 b	44.02 ± 1.12 a	29.26 ± 1.35 c	18.91 ± 0.72 d
LA	7.65 ± 0.15 c	5.48 ± 0.33 d	10.83 ± 1.70 b	15.38 ± 1.05 a
H	0.89 ± 0.03 d	5.55 ± 0.14 c	6.84 ± 0.46 b	8.88 ± 0.52 a
pH	4.15 ± 0.14 a	4.08 ± 0.04 a	4.26 ± 0.01 a	4.19 ± 0.11 a
BD (g/cm^3^)	1.12 ± 0.03 b	1.28 ± 0.04 a	1.02 ± 0.04 c	0.99 ± 0.06 c
SWC (%)	26.34 ± 0.48 b	29.07 ± 1.59 b	31.98 ± 2.37 ab	37.11 ± 2.60 a
SOC (mg/g)	28.42 ± 1.41 b	26.02 ± 1.78 b	38.62 ± 3.38 ab	45.11 ± 7.70 a
TN (mg/g)	0.48 ± 0.05 b	0.44 ± 0.05 b	0.47 ± 0.05 b	0.69 ± 0.06 a
TP (mg/g)	0.25 ± 0.00 b	0.23 ± 0.01 b	0.22 ± 0.02 b	0.34 ± 0.05 a
I_n_ (mm)	-	37.65 ± 3.14 b	46.84 ± 4.95 a	53.91 ± 3.26 a
MWC_litter_ (t/hm^2^)	1.79 ± 0.10 b	7.28 ± 0.74 a	7.66 ± 0.49 a	9.01 ± 1.41 a
MWC_soil_ (t/hm^2^)	4137 ± 169 a	4080 ± 48 a	4490 ± 170 a	4133 ± 70 a
Soil surface runoff (m^3^/hm^2^·a)	57.26 ± 28.06 b	363.18 ± 155.55 a	63.16 ± 61.60 b	2.70 ± 1.33 c
Soil erosion (t/hm^2^·a)	14.04 ± 7.99 b	61.47 ± 26.05 a	10.11 ± 9.84 b	0.26 ± 0.17 c

Values are M ± SE. Different letters represent significant differences between the restoration community types by LSD test (*p* < 0.05).

**Table 2:** Spearman correlations between plant traits and soil basic physicochemical properties and ecosystem function parameters.

	LDMC	SLA	LA	H	LNC	SRL
pH	0.420	−0.455	0.350	0.322	−0.238	−0.448
SWC	0.881 **	−0.622 *	0.483	0.769 **	−0.350	−0.615 *
BD	−0.706 *	0.895 **	−0.678 *	−0.720 **	0.238	0.860 **
SOC	0.692 *	−0.804 **	0.671 *	0.622 *	−0.077	−0.797 **
TN	0.175	−0.559	0.420	0.308	0.287	−0.545
TP	0.182	−0.594 *	0.538	0.231	0.406	−0.573 *
I_n_	0.700 *	−0.717 *	0.700 *	0.767 *	−0.083	−0.733 *
MWC_litter_	0.720 **	−0.392	0.210	0.650 *	−0.517	−0.357
MWC_soil_	0.126	−0.182	0.014	0.084	−0.196	−0.210
Soil surface runoff	−0.469	0.720 **	−0.538	−0.531	0.231	0.692 *
Soil erosion	−0.559	0.783 **	−0.608 *	−0.587 *	0.252	0.755 **

* and ** represent the significant correlation at 0.05 and 0.01 levels.

**Table 3:** Plant functional traits and the identification of two plant functional response types (PF_re_Ts).

PF_ef_T	Number of Species	Functional Traits		Presence (%)	Average Performance	Representative Species
		SLA	LNC			
1	20	2	3	100	26.5	*Lithocarpus polystachya*, *Eurya nitida*, *Osteomeles schwerinae*, *Eucalyptus smithii*
2	17	2	2	100	13	*Keteleeria evelyniana*, *Pyrus pashia*, *Ternstroemia gymnanthera*, *Myrsine africana*

SLA, specific leaf area, 1 = 0–7 m^2^/kg, 2 = 7–14 m^2^/kg, 3 = 14–22 m^2^/kg, 4 = >22 m^2^/kg; LNC, leaf nitrogen concentration, 1 = 0–10 mg/g, 2 = 10–15 mg/g, 3 = 15–20 mg/g, 4 = >20 mg/g.

**Table 4:** Plant traits and the identification of plant functional effect types (PF_ef_Ts).

PF_ef_T	Number of Species	Functional Traits			Presence (%)	Average Performance	Representative Species
		SLA	LS	SRL			
1	9	3	1	3	100	8.3	*Keteleeria evelyniana*, *Lithocarpus polystachya*
2	11	2	2	2	100	15.5	*Cyclobalanopsis glaucoides*, *Pinus yunnanensis*
3	5	1	1	2	100	6.8	*Pyrus pashia*, *Myrica nana*
4	5	2	1	4	93.3	7	*Myrsine africana*
5	2	2	5	3	33.3	3.4	*Dichotomanthus tristaniaecarpa*
6	1	1	3	4	6.7	3	*Lithocarpus dealbatus*
7	1	3	5	2	20	8	*Eucalyptus smithii*

SLA, specific leaf area, 1 = 0–7 m^2^/kg, 2 = 7–14 m^2^/kg, 3 = 14–22 m^2^/kg, 4 = >22 m^2^/kg; LS, leaf size, 1 = 0–10 cm^2^, 2 = 10–20 cm^2^, 3 = 20–30 cm^2^, 4 = 30–40 cm^2^, 5 = >40 cm^2^; SRL, specific root length, 1 = 0–15 m^2^/g, 2 = 15–30 m^2^/g, 3 = 30–45 m^2^/g, 4 = >45 m^2^/g.

**Table 5:** Redundancy analysis evaluating the relationships between PF_re_Ts and PF_ef_Ts.

Canonical Axes				Non-Canonical Axes			
I	II	III	IV	I	II	III	IV
0.153	0.060	0.003	0.001	0.308	0.279	0.243	0.170

## Data Availability

Not applicable.
